# Mr. Peter Hubert Desvignes

**Published:** 1910-07-30

**Authors:** 


### Obituary.

Mr. Peter Hubert Desvignes, M.R.C.S., late of Lewisham, has died at Weybridge at the age of seventy-three.

